# Supplementary material for: An open-source framework for large-scale, flexible evaluation of biomedical text mining systems
Source: J Biomed Discov Collab. 2008 Jan 29;3:1. doi: 10.1186/1747-5333-3-1 (PMC2276192; doi:10.1186/1747-5333-3-1)
Supplement: Additional file 1 — Evaluation platform source code. The additional file contains the software evaluation framework discussed in this paper. The most current release can be downloaded from SourceForge.net [1]. See the accompanying README file for instructions on installation and use. Also included in the distribution is a collection of UIMA wrappers for some commonly used BioNLP tools and annotated corpora. [file 1747-5333-3-1-S1.zip › bionlp_uima_repository-1.0.1/docs/api/src-html/edu/uchsc/ccp/uima/ae/nlp/sentenceDetector/LingPipeSentenceDetector_AE.html]

```
001    package edu.uchsc.ccp.uima.ae.nlp.sentenceDetector;
002    
003    import java.util.ArrayList;
004    import java.util.Iterator;
005    import java.util.List;
006    
007    import org.apache.uima.analysis_engine.ResultSpecification;
008    import org.apache.uima.analysis_engine.annotator.AnnotatorProcessException;
009    import org.apache.uima.analysis_engine.annotator.JTextAnnotator_ImplBase;
010    import org.apache.uima.cas.CASException;
011    import org.apache.uima.jcas.JCas;
012    
013    import edu.uchsc.ccp.uima.annotation.CCPDocumentInformation;
014    import edu.uchsc.ccp.uima.annotation.CCPTextAnnotation;
015    import edu.uchsc.ccp.uima.annotation.syntactic.CCPSentenceAnnotation;
016    import edu.uchsc.ccp.uima.util.UIMA_Util;
017    import edu.uchsc.ccp.util.external.lingpipe.LingPipe_Util;
018    import edu.uchsc.ccp.util.nlp.annotation.TextAnnotation;
019    import edu.uchsc.ccp.util.nlp.mention.ClassMentionTypes;
020    
021    public class LingPipeSentenceDetector_AE extends JTextAnnotator_ImplBase {
022    
023        public void process(JCas jcas, ResultSpecification arg1) throws AnnotatorProcessException {
024            /* get document text */
025            String docText = jcas.getDocumentText();
026    
027            Iterator docInfoIter = jcas.getJFSIndexRepository().getAnnotationIndex(CCPDocumentInformation.type).iterator();
028            String docID = "-1";
029            if (docInfoIter.hasNext()) {
030                CCPDocumentInformation srcDocInfo = (CCPDocumentInformation) docInfoIter.next();
031                docID = srcDocInfo.getDocumentID();
032            }
033    
034            /* parse into sentences using LingPipe Sentence Chunker */
035            List<TextAnnotation> sentenceAnnotations = new ArrayList<TextAnnotation>();
036            if (docText != null) {
037                sentenceAnnotations = LingPipe_Util.findSentences(docText, docID);
038                /* add protein annotations to the CAS */
039                UIMA_Util uimaUtil = new UIMA_Util();
040                uimaUtil.putTextAnnotationsIntoJCas(jcas, sentenceAnnotations);
041    
042                /* convert annotations with class mention name = "sentence" to CCPSentenceAnnotation */
043                Iterator textAnnotIter = jcas.getJFSIndexRepository().getAnnotationIndex(CCPTextAnnotation.type).iterator();
044                List<CCPTextAnnotation> syntacticAnnotationsToRemove = new ArrayList<CCPTextAnnotation>();
045                List<CCPTextAnnotation> syntacticAnnotationsToAdd = new ArrayList<CCPTextAnnotation>();
046    
047                while (textAnnotIter.hasNext()) {
048                    CCPTextAnnotation ccpTA = (CCPTextAnnotation) textAnnotIter.next();
049    
050                    String mentionName = ccpTA.getClassMention().getMentionName();
051    
052                    if (mentionName.equals(ClassMentionTypes.SENTENCE)) {
053                        /* if it's already a CCPSentenceAnnotation, then we don't have to do anything */
054                        if (!(ccpTA instanceof CCPSentenceAnnotation)) {
055                            CCPSentenceAnnotation sentenceAnnotation = new CCPSentenceAnnotation(jcas);
056                            try {
057                                UIMA_Util.swapAnnotationInfo(ccpTA, sentenceAnnotation);
058                            } catch (CASException ce) {
059                                ce.printStackTrace();
060                            }
061    
062                            /* remove the old annotation from the JCas, and add the new one */
063                            // ccpTA.removeFromIndexes();
064                            syntacticAnnotationsToRemove.add(ccpTA);
065                            // clauseAnnotation.addToIndexes();
066                            syntacticAnnotationsToAdd.add(sentenceAnnotation);
067                        }
068                    }
069                }
070    
071                /* add the new syntactic annotations (must add outside of while loop to avoid concurrent modification exception */
072                for (CCPTextAnnotation ccpTA : syntacticAnnotationsToAdd) {
073                    ccpTA.addToIndexes();
074                }
075    
076                /* Removed the now unnecessary SyntacticAnnotations */
077                for (CCPTextAnnotation ccpTA : syntacticAnnotationsToRemove) {
078                    ccpTA.removeFromIndexes();
079                }
080    
081            } else {
082                System.err.println("Null doctext for docid: " + docID);
083            }
084    
085        }
086    
087    }
```
